# Using bifactor exploratory structural equation modeling to examine global and specific factors in measures of sports coaches' interpersonal styles

**DOI:** 10.3389/fpsyg.2015.01303

**Published:** 2015-09-01

**Authors:** Andreas Stenling, Andreas Ivarsson, Peter Hassmén, Magnus Lindwall

**Affiliations:** ^1^Department of Psychology, Umeå UniversityUmeå, Sweden; ^2^Center of Research on Welfare, Health and Sport (CVHI), Halmstad UniversityHalmstad, Sweden; ^3^Faculty of Health, Research Institute for Sport and Exercise, University of CanberraCanberra, ACT, Australia; ^4^Department of Psychology, University of GothenburgGothenburg, Sweden; ^5^Department of Food and Nutrition, and Sport Science, University of GothenburgGothenburg, Sweden

**Keywords:** controlling behaviors, dimensionality, leadership, need support, self-report scales

## Abstract

In the present work we investigated distinct sources of construct-relevant psychometric multidimensionality in two sport-specific measures of coaches' need-supportive (ISS-C) and controlling interpersonal (CCBS) styles. A recently proposed bifactor exploratory structural equation modeling (ESEM) framework was employed to achieve this aim. In Study 1, using a sample of floorball players, the results indicated that the ISS-C can be considered as a unidimensional measure, with one global factor explaining most of the variance in the items. In Study 2, using a sample of male ice hockey players, the results indicated that the items in the CCBS are represented by both a general factor and specific factors, but the subscales differ with regard to the amount of variance in the items accounted for by the general and specific factors. These results add further insight into the psychometric properties of these two measures and the dimensionality of these two constructs.

## Introduction

Coaches' interpersonal styles strongly influence athletes' need satisfaction and motivation in competitive sports (Mageau and Vallerand, 2003). Whereas a need-supportive interpersonal style generally has a positive influence on athletes' motivation, well-being, and performance (Hagger and Chatzisarantis, 2007), a controlling interpersonal style has instead been related to maladaptive outcomes, such as burnout, depression, and disordered eating (Bartholomew et al., 2011). These interpersonal styles are multidimensional constructs, each consisting of theoretically distinguishable subdimensions. Specifically, a need-supportive interpersonal style reflects a global construct as well as three specific subdimensions: *autonomy support, structure*, and *involvement*. Similarly, a controlling interpersonal style reflects a global construct and four specific subdimensions: *controlling use of rewards, negative conditional regard, intimidation*, and *excessive personal control*. Hence, two distinct sources of psychometric multidimensionality exist: a global factor and specific subdimensions. A comprehensive test of the multidimensional structure thereby requires consideration of both sources (Morin et al., 2015).

The subdimensions of need-support measures (e.g., related to parents, coaches, or exercise practitioners) are suggested to be highly interrelated, and these measures are often treated as unidimensional (e.g., Ryan, 1991; Niemiec et al., 2006; Markland and Tobin, 2010). Given the multidimensional nature of a need-supportive interpersonal style (Deci and Ryan, 2000; Mageau and Vallerand, 2003), as well as recent suggestions that more attention should be given to these various need-supportive interpersonal styles within physical activity settings (e.g., Pope and Wilson, 2012; Standage, 2012), an investigation of the multidimensionality in measures of this construct within the sports context seems warranted. The interpersonal supportiveness scale-coach (ISS-C; Wilson et al., 2009) represents one of the few attempts to develop a multidimensional—autonomy support, structure, and involvement—sport-specific measure in line with the tenets of the self-determination theory (SDT; Deci and Ryan, 2000; Mageau and Vallerand, 2003). One concern has been, however, the relatively high degree of overlap (i.e., common variance) between the three dimensions (Wilson et al., 2009). This concern in turn has raised questions related to the instrument's dimensionality and whether global and/or specific factors are captured by the ISS-C.

When examining the factorial structure of the controlling coach behaviors scale (CCBS), which assesses athletes' perceptions of their coach's controlling interpersonal style from the perspective of SDT (Deci and Ryan, 1985, 2000), Bartholomew et al. (2010) concluded “that a controlling interpersonal style is a multidimensional construct represented by a number of separate, but related, controlling coaching strategies” (p. 205). This conclusion was reached after inspecting and comparing model fit between a four-factor first-order independent cluster model (ICM) confirmatory factor analysis (CFA), a second-order ICM-CFA with four first-order factors, and a one-factor first-order model. This approach to some extent examined multidimensionality; however, it did not identify distinct sources of construct-relevant psychometric multidimensionality in terms of simultaneous estimation of global and specific constructs, as proposed by Morin et al. (2015). Although other studies have examined the psychometric properties of CCBS (e.g., Castillo et al., 2014), a comprehensive test of the structure of the multidimensionality, taking both the global construct and specific subdimensions into consideration, has not yet been performed.

The present studies contribute to the existing literature by investing distinct sources of construct-relevant psychometric multidimensionality in these two sport-specific measures of coaches' need-supportive (ISS-C; Wilson et al., 2009) and controlling interpersonal (CCBS; Bartholomew et al., 2010) styles.

## Need-supportive interpersonal style

Previous research examining coaches' interpersonal styles in competitive sports contexts (and other contexts—see Ng et al., 2012) have primarily focused on the effects of perceived autonomy support (e.g., Adie et al., 2012; Stenling et al., 2015) and the perceived motivational climate (e.g., Sarrazin et al., 2002; Reinboth and Duda, 2006). Mageau and Vallerand (2003), however, argued that, in addition to autonomy support, coaching behaviors that provide structure and involvement are also important determinants for the satisfaction of athletes' psychological needs and their behavioral regulations. Coaches who provide autonomy support try to understand an athlete's perspective, acknowledge the athlete's feelings, encourage exploration, and curiosity, provide a meaningful rationale, and provide opportunities for choice (Mageau and Vallerand, 2003). Structure involves providing clear and understandable guidelines and expectations, instilling a sense of competence in the athletes, and providing relevant feedback to the athletes (Reeve and Su, 2014). Involvement is displayed when coaches show a genuine interest in their athletes and their well-being and spend a considerable amount of time, energy, and resources on them (Grolnick and Ryan, 1989). Need-supportive environments providing autonomy support, structure, and involvement have been examined in physical education settings (Standage et al., 2005; Haerens et al., 2013) and exercise settings (e.g., Markland and Tobin, 2010), but the potential role of perceived structure and involvement from coaches in sports is still largely unexplored (Mageau and Vallerand, 2003; Pope and Wilson, 2012). Ryan (1991) and others (e.g., Niemiec et al., 2006; Markland and Tobin, 2010) have argued the three support dimensions are highly interrelated; they are therefore often combined into a broader category labeled *need support*.

## Controlling interpersonal style

Controlling coaches may actively thwart athletes' basic psychological need satisfaction, and this has been linked to ill-being and stress responses (Bartholomew et al., 2011; Taylor et al., 2015). Based on an extensive literature review, Bartholomew et al. (2009, 2010) identified a number of controlling motivational strategies. The most prominent was *controlling use of rewards*, which refers to the use of extrinsic rewards and praise to ensure athlete compliance, engagement, and persistence in certain behaviors. This controlling strategy is closely related to the undermining effect of rewards on intrinsic motivation (Deci et al., 1999; Vansteenkiste and Deci, 2003), which refers to the negative effect of tangible rewards (task and performance contingent) on intrinsic motivation, particularly when the reward is expected. Similar effects can also be produced by verbal rewards and praise (Henderlong and Lepper, 2002).

When athletes do not display the desired attributes or behaviors, coaches may withhold attention and affection, labeled *negative conditional regard* (Assor et al., 2004). Conditional regard from coaches is displayed when their attention and acceptance is highly contingent upon athletes showing appropriate thoughts and behaviors, which oftentimes forces the athletes to give up their autonomy to maintain a satisfactory relationship with their coach (Bartholomew et al., 2010).

An abusive power-based controlling motivational strategy is *intimidation*, used to belittle and humiliate through verbal abuse, threats, and yelling to control athletes' behaviors and promote external regulation (Bartholomew et al., 2010). Such controlling strategies create pressure from the outside, which promotes athletes to engage in certain behaviors to avoid external punishment (Deci and Ryan, 1987).

Finally, *excessive personal control* is displayed when coaches engage in intrusive monitoring of athletes' free time and impose strict limits (Bartholomew et al., 2010). Examples of such coach behaviors involve restricting athletes' free time (e.g., setting curfews) or engagement in other sports. This type of controlling interpersonal behavior promotes a sense of pressure from the coach to prioritize one's sports involvement over other important aspects of the athlete's life.

## The present studies

The two instruments under scrutiny here, the ISS-C (Wilson et al., 2009) and the CCBS (Bartholomew et al., 2010), contain three and four subscales, respectively, and can both be described in terms of a broader construct as well as more specific dimensions or subdomains within that broader construct. Hence, both these instruments are suited for a comprehensive examination of these two sources of construct-relevant variance. One particularly useful approach for this aim, which recently has been rediscovered within psychology research, is the bifactor measurement model (Reise, 2012). The bifactor measurement model originates from the early work by Holzinger and Swineford (1937) but has for a long time been overshadowed by Thurstone's (1947) correlated-factor model. The bifactor model has not only been rediscovered but also extended within an ESEM framework (Jennrich and Bentler, 2011, 2012; Myers et al., 2014; Morin et al., 2015).

Theory-based multidimensional scales, such as the ISS-C and CCBS, often correspond to a bifactor structure with a general latent construct alongside several latent subdimensions that are more narrowly defined (Myers et al., 2014). Applications of bifactor measurement models, however, are rare in sport and exercise psychology research, despite the fact that a bifactor model often provides researchers with an opportunity to match the theory behind the instrument development with the model imposed on the data when evaluating multidimensional scales. This match between theory and model may be lacking when the commonly used correlated first-order model or a second-order factor model is specified because neither of these two models takes the general latent constructs' direct influence on items into account (Myers et al., 2014).

In addition to the rediscovered bifactor measurement model (Reise, 2012), the recently developed ESEM allows researchers to deal with a common problem with ICM-CFA, namely the fallible nature of indicators (Morin et al., 2015). Items incorporate a part of random measurement error, also known as item uniqueness, but items also tend to have some degree of systematic association with other constructs. Such systematic association is typically expressed as cross-loadings in exploratory factor analysis but is constrained to zero in ICM-CFA. In the context of theory-driven multidimensional scales, this assumption of zero cross-loadings might be unrealistically restrictive and, thus, lead to extensive bias in factor correlations and poor model fit (e.g., Asparouhov and Muthén, 2009; Marsh et al., 2014). Multidimensional measures typically include cross-loadings that can be justified by substantive theory or item content (Asparouhov and Muthén, 2009); most items are likely to be imperfect to some degree and have some systematic association with other constructs (Morin et al., 2015).

The recent incorporation of bifactor models and ESEM provides researchers with an opportunity to investigate two sources of “construct-relevant psychometric multidimensionality related to: (a) the hierarchical nature of the constructs being assessed (i.e., the co-existence of global and specific components within the same measurement model) and (b) the fallible nature of indicators which tend to include at least some degree of association with non-target constructs” (Morin et al., 2015, p. 30). According to Morin and colleagues, bifactor models are needed to investigate the first source, whereas the second source calls for ESEM rather than CFA. Furthermore, by estimating ESEM with target rotation, it is possible to specify a priori hypotheses about the factor structure and use ESEM for confirmatory purposes (Asparouhov and Muthén, 2009).

The purpose of the two studies outlined in this report was to apply the recently proposed bifactor ESEM framework (Morin et al., 2015) and investigate distinct sources of construct-relevant psychometric multidimensionality in two sport-specific measures of coaches need-supportive and controlling behaviors: the ISS-C (Wilson et al., 2009) and the CCBS (Bartholomew et al., 2010). Because these two instruments are expected to consist of a general latent factor alongside several narrowly defined subdimensions, we hypothesized that the first-order ESEM would provide a better fit to the data compared to the first-order ICM-CFA and that the bifactor ESEM would provide a better fit to the data compared to first-order ESEM.

## Study 1—interpersonal supportiveness scale–coach

### Materials and methods

#### Participants

The sample comprised 277 (142 female, 135 male) floorball players competing for clubs in northern Sweden. The athletes' ages ranged from 15 to 22 years (*M* = 16.8, *SD* = 1.1) and their competitive levels from regional to international. On average, they practiced floorball 6.8 h (*SD* = 3.4) per week and had been competing in their sport for 8.4 years (*SD* = 2.8).

#### Measures

We used a Swedish version of the Interpersonal Supportiveness Scale–Coach (ISS–C; Wilson et al., 2009) to capture athletes' perceptions of their coach's autonomy support (six items, e.g., “My coach provides me with choices and options”), provision of structure (six items, e.g., “My coach provides clear feedback about my progress”), and involvement (six items, e.g., “My coach puts time and energy into helping me”). Responses to the 18 items were given on a 7-point Likert scale ranging from 1 (*strongly disagree*) to 7 (*strongly agree*). Internal consistency (omega coefficient; McDonald, 1999) of the three subscales was as follows: autonomy support = 0.860, structure = 0.902, involvement = 0.781.

#### Statistical analysis

Data were analyzed with Mplus version 7.3 (Muthén and Muthén, 1998–2012) and the robust maximum likelihood estimator (MLR). MLR provides standard errors and fit indexes that are robust to the Likert nature of the items and non-normality. The items were treated as continuous variables. In a recent simulation study Rhemtulla et al. (2012) showed that the robust maximum likelihood estimator performs equally well or better compared to robust categorical estimators, particularly with seven response categories. A small percentage of missing data was present in the items (< 2.2%). We choose to include all available information and used the full information robust maximum likelihood (FIML) estimation to handle the missing data (Enders, 2010).

**Figure 1:**
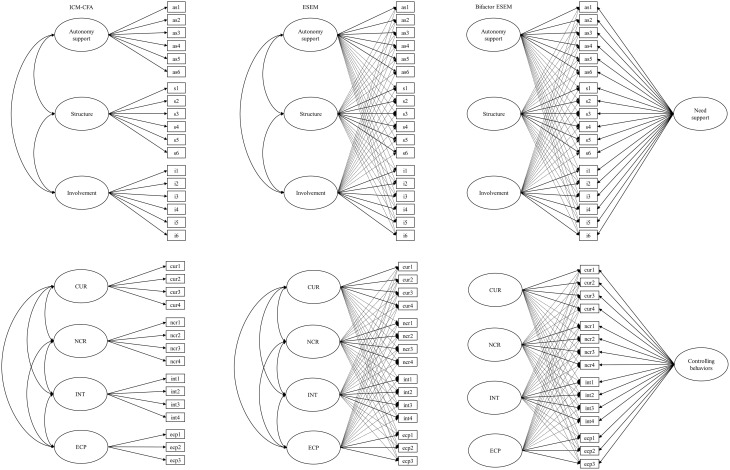
**Graphical representation of the alternative models tested in these two studies**. The top three are the ICM-CFA, first-order ESEM, and bifactor ESEM of the ISS-C. Bottom three are the ICM-CFA, first-order ESEM, and bifactor ESEM of the CCBS. Dotted lines represent non-target loadings. CUR, controlling use of rewards; NCR, negative conditional regard; INT, intimidation; and ECP, excessive personal control.

We used a model testing procedure proposed by Morin et al. (2015). This procedure allowed us to investigate two sources of construct-relevant psychometric multidimensionality related to the co-existence of global and specific components within the same measurement model and the fallible nature of indicators which tend to include at least some degree of association with non-target constructs. We started by specifying and comparing first-order ICM–CFA with first-order ESEM models to examine the presence of cross-loadings of conceptually related or overlapping constructs. Based on the results in the first step (ICM–CFA vs. ESEM), the second step aimed to identify the presence of construct-relevant multidimensionality due to the presence of hierarchically superior constructs using bifactor models. The ICM–CFA, ESEM, and bifactor ESEM models are graphically depicted in Figure 1,^1^.

Conventional fit indices were used to evaluate the model fit in the ICM-CFA and ESEM models, such as the comparative fit index (CFI), the Tucker-Lewis Index (TLI), the standardized root mean residual (SRMR), and the root mean square error of approximation (RMSEA). Traditional cutoff criteria with CFI and TLI values around 0.90 and SRMR and RMSEA values around 0.08 were used to indicate acceptable fit (Marsh, 2007). Target rotation was used in the ESEM models, which allows for the specification of target and non-target factor loadings in a confirmatory manner (Browne, 2001; Asparouhov and Muthén, 2009). All cross-loadings were specified to be close to zero, while all the main loadings were freely estimated (Morin et al., 2015). In line with bifactor assumptions that the specific factors explains item variance not explained by the general factor and that the general factor explains variance that is shared across all items, the general and specific factors were orthogonal in order to ensure interpretability (Chen et al., 2006; Reise, 2012). The first-order ESEM models were estimated with an oblique target rotation (Browne, 2001; Asparouhov and Muthén, 2009).

We followed the guidelines for nested model comparisons in small samples (< 300) by Chen (2007), who suggested that a change in CFI (ΔCFI) ≥ −0.005 accompanied by a change in RMSEA (ΔRMSEA) of ≥ 0.010 would support the simpler model over the more complex model. In addition, Marsh et al. (2010) suggested that some indices (e.g., TLI and RMSEA) penalize for parsimony so that a more parsimonious model can fit the data better than a less parsimonious model. Therefore, a more conservative approach is to consider the more parsimonious model as supported, if the TLI or RMSEA is as good or better compared to the more complex model. We also used several information criteria when comparing alternative models: the Akaike Information Criterion (AIC; Akaike, 1987), the Bayesian Information Criterion (BIC; Schwartz, 1978), and the sample-size adjusted BIC (ABIC; Sclove, 1987). These information criteria do not in themselves describe model fit, but a model with a lower value indicates a better fitting model compared to a model with a higher value when alternative models are compared. Note, however, that all of these guidelines for model fit have been established for CFA, and more work is needed regarding their generalizability outside of the CFA framework. Regardless of the framework these guidelines are applied within (e.g., CFA, ESEM), they should not be taken as “golden rules” but rather as rough guidelines used in combination with parameter estimates, statistical conformity, and theoretical adequacy (Marsh et al., 2004; Morin et al., 2015).

#### Procedure

The initial contact was made with the head coach of each team, and when permission was granted to approach the athletes, a time, and place were scheduled for an informational meeting with them. During this meeting, a research assistant invited the athletes to participate in the study. Upon agreement to participate, the athletes provided written informed consent and responded to a multi-section questionnaire that took approximately 20 min to complete. Data was collected at midseason to ensure that the athletes had had enough time to establish a perception of their coach's interpersonal style. Prior to the data collection, ethical approval was obtained from the Regional Ethical Review Board at the first author's university.

### Results

Item correlations, means, standard deviations, skewness, and kurtosis are displayed in Table 1. As seen in Table 2, the first-order ICM–CFA model displayed an acceptable fit to the data. However, the first-order ESEM model displayed a better representation of the data as indicated by the ΔCFI = 0.049, ΔRMSEA = 0.018, higher TLI and lower RMSEA values, and lower AIC, BIC, and ABIC values. The ICM–CFA model displayed large factor correlations between the three factors (0.956–0.992), but these factor correlations were lower in the first-order ESEM model (0.473–0.708; see Table 3). The first-order ESEM model also revealed several cross-loading items, with relatively high loadings on non-target factors. Taken together, the ESEM model provided a better representation of the data compared to the ICM–CFA and was therefore retained in the second step when estimating the bifactor model. As displayed in Table 2, the bifactor ESEM model provided an excellent fit to the data and had lower AIC and ABIC compared to the first-order ESEM. The factor loading pattern from the bifactor ESEM model is displayed in Table 4, and almost all items had a strong standardized factor loading on the general factor and a weak loading on the specific factor. These results indicate that most of the variance in the items in the ISS-C was accounted for by the general factor.

**Table 1:** **Correlations and descriptives for the ISS-C items**.

	**AS1**	**AS2**	**AS3**	**AS4**	**AS5**	**AS6**	**S1**	**S2**	**S3**	**S4**	**S5**	**S6**	**I1**	**I2**	**I3**	**I4**	**I5**	**I6**
AS1	–																	
AS2	0.667	–																
AS3	0.505	0.449	–															
AS4	0.553	0.553	0.439	–														
AS5	0.423	0.437	0.461	0.433	–													
AS6	0.568	0.666	0.441	0.540	0.505	–												
S1	0.614	0.600	0.435	0.368	0.380	0.598	–											
S2	0.480	0.618	0.360	0.373	0.378	0.537	0.511	–										
S3	0.504	0.546	0.520	0.446	0.411	0.500	0.584	0.477	–									
S4	0.571	0.665	0.496	0.584	0.471	0.763	0.585	0.575	0.530	–								
S5	0.561	0.593	0.498	0.505	0.548	0.674	0.709	0.550	0.602	0.682	–							
S6	0.534	0.584	0.482	0.485	0.481	0.694	0.678	0.484	0.562	0.669	0.723	–						
I1	0.671	0.731	0.440	0.500	0.398	0.643	0.696	0.490	0.564	0.657	0.621	0.626	–					
I2	0.238	0.405	0.175	0.258	0.219	0.377	0.380	0.345	0.270	0.347	0.364	0.333	0.428	–				
I3	0.517	0.592	0.536	0.569	0.474	0.601	0.605	0.503	0.611	0.662	0.663	0.659	0.637	0.361	–			
I4	0.317	0.369	0.212	0.382	0.237	0.333	0.204	0.224	0.265	0.320	0.286	0.258	0.297	0.322	0.229	–		
I5	0.546	0.581	0.533	0.532	0.501	0.755	0.658	0.515	0.520	0.693	0.748	0.691	0.637	0.294	0.692	0.270	–	
I6	0.138	0.165	0.137	0.128	0.155	0.191	0.139	0.175	0.078^a^	0.166	0.142	0.196	0.210	0.317	0.153	0.278	0.206	–
*M*	5.283	5.242	4.531	4.862	4.585	5.207	4.606	5.585	4.665	5.325	4.869	4.634	5.009	5.430	4.156	5.949	4.869	5.203
*SD*	1.382	1.535	1.661	1.472	1.400	1.506	1.731	1.400	1.453	1.465	1.506	1.613	1.661	1.402	1.463	1.298	1.493	1.453
Skew	−0.607	−0.733	−0.123	−0.254	−0.024	−0.487	−0.216	−1.051	−0.240	−0.573	−0.225	−0.237	−0.526	−0.825	0.064	−1.562	−0.303	−0.457
Kurt	−0.446	−0.178	−0.875	−0.616	−0.450	−0.689	−1.035	0.733	−0.210	−0.515	−0.823	−0.626	−0.666	0.051	−0.605	2.260	−0.596	−0.629

a *Not a statistical association at α = 0.05. AS, autonomy support, S, structure, I, involvement*.

**Table 2:** **Model Fit of the ICM-CFA and ESEM Models**.

	**χ^2^**	***p***	***df***	**CFI**	**TLI**	**SRMR**	**RMSEA [90% CI]**	**AIC**	**BIC**	**ABIC**
**STUDY 1**										
First-order ICM-CFA	354.736	<0.001	132	0.910	0.895	0.048	0.078 [0.068, 0.088]	15228.673	15435.242	15254.503
First-order ESEM	202.779	<0.001	102	0.959	0.939	0.036	0.060 [0.048, 0.072]	15092.606	15407.896	15132.031
Bifactor ESEM	155.273	<0.001	87	0.972	0.951	0.030	0.053 [0.039, 0.067]	15055.051	15424.701	15101.273
**STUDY 2**										
First-order ICM-CFA	146.495	<0.001	84	0.927	0.908	0.061	0.057 [0.041, 0.072]	10600.452	10424.449	10438.807
First-order ESEM	85.079	0.002	51	0.960	0.917	0.030	0.054 [0.032, 0.073]	10404.486	10694.373	10428.134
Bifactor ESEM	51.226	0.110	40	0.987	0.965	0.020	0.035 [0.000, 0.060]	10387.989	10715.838	10414.735

**Table 3:** **Latent factor correlations between the subdimensions in the ISS-C**.

	**Autonomy support**	**Structure**	**Involvement**
Autonomy support	–	0.956	0.984
Structure	0.708	–	0.992
Involvement	0.473	0.488	–

*ICM-CFA correlations are displayed above the diagonal and first-order ESEM correlations are displayed below the diagonal*.

**Table 4:** **Standardized Loading Pattern for the Bifactor ESEM of the ISS-C (***N*** = 277)**.

**Item**	**General factor**		**Autonomy support (AS)**		**Structure (S)**		**Involvement (I)**		***R*^2^ (%)**
	**λ**	***SE***	**λ**	***SE***	**λ**	***SE***	**λ**	***SE***	
AS1	0.721	0.043	**0.203^a^**	**0.250**			0.205	0.098	61.3
AS2	0.808	0.037	**0.104^a^**	**0.247**			0.347	0.065	78.5
AS3	0.604	0.051	**0.307^a^**	**0.168**					49.8
AS4	0.674	0.046	**0.221^a^**	**0.147**	−0.213	0.064			54.9
AS5	0.594	0.048	**0.110^a^**	**0.160**					40.4
AS6	0.858	0.026	−**0.258^a^**	**0.200**					81.4
S1	0.744	0.035			**0.556**	**0.109**			87.5
S2	0.652	0.037			**0.035^a^**	**0.082**			44.1
S3	0.669	0.040	0.253	0.071	**0.196**	**0.091**			55.3
S4	0.846	0.021			−**0.083^a^**	**0.064**			72.8
S5	0.817	0.027			**0.205**	**0.077**			74.3
S6	0.795	0.029			**0.169**	**0.071**			68.4
I1	0.796	0.027					**0.244**	**0.078**	72.1
I2	0.434	0.055					**0.211^a^**	**0.138**	24.5
I3	0.780	0.030					−**0.147^a^**	**0.098**	66.1
I4	0.395	0.058					**0.151^a^**	**0.125**	21.6
I5	0.835	0.033					−**0.216^a^**	**0.121**	76.5
I6	0.233	0.063					−**0.070^a^**	**0.135**	7.2

a *Not a statistical effect at α = 0.05. For clarity in the table, cross-loadings below 0.20 are not displayed in the table (cf. Jennrich and Bentler, 2012; Myers et al., 2014). Target factor loadings are in bold*.

### Discussion

The comparison between the first-order ESEM and the bifactor ESEM indicated that the latter provided a better fit to the data. However, the first-order ESEM indicated a substantial degree of cross-loading items, and the bifactor ESEM displayed that most of the variance in the ISS–C items was accounted for by the general factor. This indicates that the ISS–C items were captured by the general construct need support and not by the specific subdimensions autonomy support, structure, and involvement. These results are similar to previous multidimensional leadership scales, for example, within transformational leadership research (e.g., van Knippenberg and Sitkin, 2013), where the subdimensions often are highly interrelated. A high degree of overlap between subdimensions within multidimensional SDT need-support scales is quite common (e.g., Niemiec et al., 2006; Wilson et al., 2009) and the three need-support dimensions are often suggested to be highly interrelated (Ryan, 1991; Markland and Tobin, 2010). Much SDT research has therefore treated need support as a unidimensional variable (e.g., only using autonomy support), and have not acknowledged the multidimensional nature of need support as consisting of autonomy support, structure, and involvement. The high correlations between the need-support dimensions could be explained by the fact that coaches who are need supportive with regard to one dimension (e.g., autonomy support) also are need supportive with regard to the other dimensions. Such an explanation has previously been suggested, for example, within transformational leadership research, where high factor correlations are commonly observed in research with self-report measures (Barling et al., 2011). Another explanation is related to the use of self-report measures and that the respondents may not be able to distinguish between items intended to capture these various dimensions of leadership. A remedy for this type of common method bias could be to collect other types of data (Podsakoff et al., 2012), such as observational data of coach behaviors in various situations. SDT-based observations (e.g., Haerens et al., 2013; Smith et al., 2015) could be a useful source for data collection to avoid common method bias when combined with other data sources (e.g., self-report).

Having measures that can discriminate between subdimensions in multidimensional constructs is important because this would allow researchers to explore whether these leadership dimensions are additive (i.e., the more, the better), which is assumed when sum scores of need support are used or if their relationships have other forms (van Knippenberg and Sitkin, 2013). For example, instead of assuming an additive effect, we could specify a minimum value on each of these dimensions that determines when someone can be characterized as need supportive. The need-support dimensions could also be interactive, indicating that the engagement in one type of need support would make the other types more effective. This type of moderating effect has been found in educational research (Jang et al., 2010) where autonomy support and structure were two interacting engagement-fostering interpersonal styles among teachers associated with students' classroom engagement. A third possibility could be that one need-support dimension can compensate for the lack of another (van Knippenberg and Sitkin, 2013). The evidence so far suggests that the three need-support dimensions are interactive, in that structure, and involvement will be enhanced when provided in an autonomy-supportive way (Reeve and Su, 2014). In order to investigate these various hypotheses, it is imperative that the three need-support dimensions are distinguishable. The multidimensionality in need-support instruments is an important avenue of future research.

## Study 2—coaches controlling behaviors scale

### Materials and methods

#### Participants

This sample comprised 233 male ice hockey players competing for clubs in northern Sweden. Their ages ranged from 15 to 20 years (*M* = 17.1, *SD* = 1.4) and their competitive levels from regional to international. On average, they had been competing in their sport for 10.6 years (*SD* = 2.1).

#### Measures

A Swedish version of the Controlling Coach Behaviors Scale (CCBS; Bartholomew et al., 2010) was used to measure the athletes' perceptions of their coach's controlling interpersonal style. The CCBS consists of four subscales capturing athletes' perceptions of coaches' controlling use of rewards (four items, e.g., My coach tries to motivate me by promising to reward me if I do well), negative conditional regard (four items, e.g., My coach is less supportive of me when I am not training and competing well), intimidation (four items, e.g., My coach intimidates me into doing the things that he/she wants me to do), and excessive personal control (three items, e.g., My coach tries to interfere in aspects of my life outside of my sport). Responses to the 15 items were given on a 7-point Likert scale ranging from 1 (*strongly disagree*) to 7 (*strongly agree*). Internal consistency (omega coefficient; McDonald, 1999) of the four subscales was as follows: controlling use of rewards = 0.723, negative conditional regard = 0.865, intimidation = 0.719, and excessive personal control = 0.706.

#### Statistical analysis

The same statistical analyses as in Study 1 were conducted in Study 2.

#### Procedure

The same procedure as in Study 1 was used in Study 2.

### Results

Item correlations, means, standard deviations, skewness, and kurtosis are displayed in Table 5. The first-order ICM–CFA displayed an acceptable fit to the data (Table 2), but also for the CCBS the first-order ESEM model provided a better representation of the data as indicated by the ΔCFI = 0.033, higher TLI value, and lower AIC and ABIC values. The ΔRMSEA did not reach Chen's (2007) recommendation of 0.010 but was lower for the ESEM model, and taken together, we concluded that the ESEM model provided a better representation of the data. As seen in Table 6, the factor correlations were slightly higher in the ICM–CFA model (0.149–0.597) compared to the first-order ESEM model (0.131–0.476). No substantive cross-loadings were observed in the first-order ESEM, the largest cross-loading was −0.26 (standardized). As seen in Table 2, the bifactor ESEM displayed an excellent fit to the data and had lower AIC and ABIC compared to the first-order ESEM. The factor loading pattern from the bifactor ESEM model is displayed in Table 7. The factor loadings of items in two of the specific factors, negative conditional regard and excessive personal control, indicate that the items in both these factors are explained by the general factor as well as their specific factors. Items on the subscale controlling use of rewards displayed relatively weak loadings on the general factor and strong loadings on the specific factor, whereas items on the subscale intimidation displayed an opposite pattern with stronger loadings in the general factor and weak loadings on the specific factor. These results indicate that the items in the CCBS are represented by a general factor and specific factors but that the subscales differ with regard to the amount of variance accounted for by the general and specific factors.

**Table 5:** **Correlations and descriptives for the CCBS items**.

	**CUR1**	**CUR2**	**CUR3**	**CUR4**	**NCR1**	**NCR2**	**NCR3**	**NCR4**	**INT1**	**INT2**	**INT3**	**INT4**	**EPC1**	**EPC2**	**EPC3**
CUR1	–														
CUR2	0.204	–													
CUR3	0.392	0.502	–												
CUR4	0.317	0.421	0.683	–											
NCR1	0.036^a^	0.213	0.275	0.316	–										
NCR2	0.002^a^	0.202	0.341	0.397	0.612	–									
NCR3	0.026^a^	0.185	0.305	0.386	0.623	0.676	–								
NCR4	−0.024^a^	0.170	0.280	0.294	0.562	0.507	0.665	–							
INT1	0.059^a^	0.052^a^	0.087^a^	−0.025^a^	0.389	0.334	0.268	0.324	–						
INT2	0.078^a^	0.131	0.188	−0.017^a^	0.204	0.186	0.236	0.255	0.326	–					
INT3	0.055^a^	0.125	0.157	0.061^a^	0.233	0.237	0.336	0.272	0.348	0.442	–				
INT4	0.162	0.048^a^	0.087^a^	0.030^a^	0.317	0.287	0.282	0.212	0.521	0.278	0.407	–			
EPC1	−0.051^a^	0.143	0.189	0.185	0.426	0.298	0.380	0.378	0.357	0.210	0.146	0.254	–		
EPC2	−0.026^a^	0.129	0.128^a^	0.092^a^	0.374	0.237	0.286	0.239	0.377	0.228	0.230	0.286	0.468	–	
EPC3	0.049^a^	0.090^a^	0.010^a^	0.028^a^	0.288	0.143	0.194	0.180	0.314	0.142	0.232	0.341	0.290	0.583	–
*M*	2.289	2.816	1.952	1.783	2.138	2.377	2.528	2.260	1.627	1.323	1.180	1.249	2.339	1.819	1.672
*SD*	1.671	1.821	1.333	1.218	1.626	1.731	1.644	1.418	1.251	0.883	0.609	0.693	1.699	1.239	1.102
Skew	1.008	0.779	1.311	1.634	1.521	1.222	0.972	0.931	2.468	3.797	4.101	4.068	1.272	1.827	1.964
Kurt	−0.145	−0.442	0.738	2.133	1.510	0.512	0.075	−0.110	6.077	16.866	18.124	22.669	0.755	3.360	4.334

a *Not a statistical association at α = 0.05. CUR, controlling use of rewards; NCR, negative conditional regard; INT, intimidation, EPC, excessive personal control*.

**Table 6:** **Latent factor correlations between the subdimensions in the CCBS**.

	**CUR**	**NRC**	**INT**	**EPC**
CUR	–	0.461	0.149	0.164
NCR	0.394	–	0.552	0.472
INT	0.131	0.368	–	0.597
EPC	0.131	0.417	0.476	–

*ICM-CFA correlations are displayed above the diagonal and first-order ESEM correlations are displayed below the diagonal. CUR, controlling use of rewards; NCR, negative conditional regard; INT, intimidation; EPC, excessive personal control*.

**Table 7:** **Standardized Loading Pattern for the Bifactor ESEM of the CCBS (***N*** = 233)**.

**Item**	**General factor**		**Controlling use of rewards (CUR)**		**Negative conditional regard (NCR)**		**Intimidation (INT)**		**Excessive personal control (EPC)**		***R*^2^ (%)**
	**λ**	***SE***	**λ**	***SE***	**λ**	***SE***	**λ**	***SE***	**λ**	***SE***	
CUR1	0.194	0.093	**0.440**	**0.075**	−0.213	0.082					29.6
CUR2	0.183	0.089	**0.515**	**0.067**							31.6
CUR3	0.278	0.085	**0.830**	**0.057**							79.2
CUR4	0.181^a^	0.192	**0.745**	**0.079**	0.301	0.102					70.1
NCR1	0.544	0.146			**0.519**	**0.124**					59.6
NCR2	0.493	0.141			**0.574**	**0.121**					60.4
NCR3	0.516	0.075			**0.681**	**0.083**					74.4
NCR4	0.447	0.073			**0.594**	**0.066**					57.4
INT1	0.655	0.076					**0.051^a^**	**0.421**			45.8
INT2	0.468^a^	0.471					**0.597^a^**	**0.402**			57.6
INT3	0.543	0.235					**0.293^a^**	**0.537**			38.3
INT4	0.802	0.092					**−0.167^a^**	**0.888**			72.8
EPC1	0.418	0.063			0.244	0.079			**0.329**	**0.079**	34.6
EPC2	0.474	0.105							**0.787**	**0.126**	84.5
EPC3	0.446	0.171							**0.474**	**0.140**	44.2

a *Not a statistical effect at α = 0.05. For clarity in the table, cross-loadings below 0.20 are not displayed in the table (cf. Jennrich and Bentler, 2012; Myers et al., 2014). Target factor loadings are in bold*.

### Discussion

Also for the CCBS, the bifactor ESEM provided a better fit to the data compared to the first-order ESEM. The bifactor ESEM provided an interesting pattern of multidimensionality, indicating that the CCBS consists of a general factor as well as specific factors but that the subdimensions differed with regard to the amount of variance accounted for by the general and specific factors. Researchers have previously concluded that the CCBS is a multidimensional instrument using first- and second-order ICM–CFAs (e.g., Bartholomew et al., 2010; Castillo et al., 2014). Although these previous studies, to some extent, examined multidimensionality, they did not examine distinct sources of multidimensionality in terms of the simultaneous estimation of global and specific factors. By using a bifactor ESEM approach (Morin et al., 2015), we extended previous research on the factor structure of the CCBS with a statistical method that matches the theory underlying the development of the CCBS with the model imposed on the data (Myers et al., 2014).

The bifactor structure for the CCBS indicated that the *negative conditional regard* factor and the *excessive personal control* factor have a bifactor pattern with items loading relatively strong onto both the general and the specific factors. These two subdimensions seem to consist of two sources of construct-relevant multidimensionality (Morin et al., 2015). Items on the factor *controlling use of rewards* had relatively weak loadings on the general factor compared to the other factors (range 0.181–0.278) and relatively strong loadings on its specific factor (range 0.440–0.830). This pattern was also seen in the first-order ESEM with relatively low factor correlations between the *controlling use of rewards* factor and the other factors (range 0.131–0.394). These results indicate that the *controlling use of rewards* factor may represent a slightly different aspect of coaches' controlling behaviors compared to the other three specific factors that to a larger degree seem to have a common core, as represented by the general factor. Research looking at the different CCBS factors' relationship to other variables is scarce, but *controlling use of rewards* has, for example, shown a weaker relationship to autonomy support compared to the other three factors in the CCBS (Bartholomew et al., 2010). Whether the factors in the CCBS predict outcomes differently is an interesting question for future research that would provide more insight into similarities and differences between the CCBS factors. Finally, the items on the *intimidation* factor displayed relatively strong loadings onto the general factor and weak loadings onto its specific factor. Hence, this factor is mostly explained by one source of construct-relevant multidimensionality, the general factor.

Furthermore, three items displayed meaningful cross-loadings (factor loadings > 0.20) onto the negative conditional regard factor: CUR_1, My coach tries to motivate me by promising to reward me if I do well, CUR_4, My coach only uses rewards/praise so that I complete all the tasks he/she sets in training, EPC_1, My coach expects my whole life to center on my sport participation. Future research should investigate these items further and explore whether they need to be revised or if this complexity is theoretically meaningful (cf. Myers et al., 2014). It should be noted, however, that pure items are not a requirement of a well-defined factor structure and that one can argue that it is more important to find an accurate set of items rather than a pure set of items (Asparouhov and Muthén, 2009). Hypotheses about pure factor structures with items loading solely onto their intended factors may in many cases be too restrictive due to the fallible nature of indicators, and the ESEM framework provides researchers with a tool that accounts for items' associations with non-target constructs (Asparouhov and Muthén, 2009; Myers et al., 2014; Morin et al., 2015).

## General discussion and conclusions

Our purpose was to investigate distinct sources of construct-relevant psychometric multidimensionality in two sport-specific measures of coaches' need-supportive and controlling behaviors, the ISS-C (Wilson et al., 2009) and the CCBS (Bartholomew et al., 2010). We expected that these two multidimensional instruments would consist of a general latent factor alongside several narrowly defined subdimensions and therefore adopted a recently proposed bifactor ESEM approach (Morin et al., 2015) suitable for examining such multidimensional structures.

In the first step, we compared a traditional ICM–CFA with ESEM to examine the possibility that the items within these two instruments have systematic associations with non-target constructs that needs to be accounted for (Asparouhov and Muthén, 2009; Morin et al., 2015; Perry et al., 2015). In both cases did the ESEM model provide a better fit to the data compared to the ICM–CFA, and we also noted a decrease in factor correlations because the relations between the items have to a lesser degree been channeled through the factors. Previous research with simulated (e.g., Asparouhov and Muthén, 2009) and real data (e.g., Marsh et al., 2013) have consistently shown that factor correlations in ICM–CFA are likely to be positively biased and that ESEM more accurately estimates factor correlations (Marsh et al., 2014). A pattern of positively biased factor correlations was evident also in this study. When the assumption of zero cross-loadings does not hold, the factor correlations will be positively biased because systematic associations between items and non-target factors are not accounted for (Marsh et al., 2014). Marsh and colleagues also argued that when the assumption of zero cross-loadings does hold, allowing for cross-loadings is unlikely to result in negative bias in factor correlations. Recently there have been suggestions in the methodological literature that ICM–CFA and ESEM should routinely be performed on the same data in order to investigate these potential sources of bias inherent in many ICM–CFA models (Marsh et al., 2013, 2014).

The present study contributes to the growing body of knowledge regarding the factor structure of the ISS–C and the CCBS. However, some limitations exist. The samples in these two studies were in a quite narrow age range, only included team sport athletes, and only male athletes were included in Study 2. Although the CCBS have been found invariant across type of sport and gender (Bartholomew et al., 2010), exploring whether these results can be replicated in other samples of different ages and types of sports would be interesting in future research. Researchers could also examine if perceptions of coaches need supportive and controlling interpersonal styles vary with athletes' age and competitive level. Future research should also explore the predictive ability of the general and specific factors in these scales on various outcomes, for example, basic need thwarting and satisfaction (see Gunnell and Gaudreau, 2015, for such an approach). The results from Study 1, however, suggests that the ISS-C might best be represented as a unidimensional structure and that almost all of the variance in the items was accounted for by the general factor. The unidimensional structure of the ISS-C indicates that it might not be necessary to use the general factor from a bifactor ESEM model as an outcome or predictor; a single factor can be adequate for such purposes^2^.

Taken together, the present study illustrates a useful approach for examining dimensionality in multidimensional self-report scales. The results related to the ISS–C, combined with findings from previous research, indicate that the multidimensionality of the need support construct can be questioned. Future research should thoroughly examine whether this lack of multidimensionality in need-support scales can be attributed to theoretical, methodological, and/or empirical aspects. The results related to the CCBS showed an interesting pattern of psychometric multidimensionality that should be cross-validated in future research. The integration of bifactor analysis and ESEM (Myers et al., 2014; Morin et al., 2015) provide researchers with a framework uniquely suited for examinations of multidimensional constructs.

## Author contributions

AS came up with the initial idea and design, performed the analyses and interpreted the data, and was in charge of drafting the manuscript. AI made substantial contribution to the analyses and interpretation of the data, and revised the manuscript critically for important intellectual content. PH helped out in the interpretation of data and revised the manuscript critically for important intellectual content. ML made substantial contribution to the analyses and interpretation of the data, and revised the manuscript critically for important intellectual content. All four authors (AS, AI, PH, ML) provided final approval of the version to be published, and agree to be accountable for all aspects of the work in ensuring that questions related to the accuracy or integrity of any part of the work are appropriately investigated and resolved.

### Conflict of interest statement

The authors declare that the research was conducted in the absence of any commercial or financial relationships that could be construed as a potential conflict of interest.

## Footnotes

^1^We did not include second-order models in our model testing sequence because we wanted to examine the direct relation between the global and specific factors and the items. In a second-order model the relation between the general factor and the items is represented as an indirect effect through the first-order factors. In addition, second-order models rely on far more stringent assumptions than bifactor models as well as stringent proportionality constraints not likely to hold in real-world settings with complex instrument (Morin et al., 2015; Reise, 2012).

^2^To further examine this issue with the ISS-C we correlated factor scores from a single-factor CFA model and factor scores from the general factor in the bifactor ESEM model. The correlation was 0.996 indicating very little impact of ignoring multidimensionality and suggests that using the ISS-C as a single factor might be adequate.
